# The Development of Novel Treatment Strategies for Rhabdomyosarcoma

**DOI:** 10.3390/cancers18040690

**Published:** 2026-02-19

**Authors:** Kenji Nakano

**Affiliations:** Department of Medical Oncology, Cancer Institute Hospital of the Japanese Foundation for Cancer Research, Tokyo 135-8550, Japan; kenji.nakano@jfcr.or.jp

**Keywords:** rhabdomyosarcoma, pediatric cancer, FOXO1, MYOD1, RAS, ALK, NTRK, FGFR, MSI-High

## Abstract

Rhabdomyosarcoma is a small round-cell soft tissue tumor that occurs mainly in pediatric and adolescent/young adult (AYA) patients but also rarely in adults. Multidisciplinary treatments including multidrug therapy and local therapy (surgery and/or radiation) are the current standard of care, and treatment strategies are determined according to the estimated risk based on the patient’s age, site of onset, and histologic type, as well as the disease stage. New treatment developments in recent years have been based on risk; lower cumulative doses of alkylating agents to reduce late toxicity for low-risk patients are being studied, and long-term maintenance therapy or the addition of new drugs inhibitors to standard multidisciplinary therapy for intermediate- to high-risk patients have been investigated. For high-risk and metastatic patients, novel molecular targeted drug candidates are being evaluated. The target candidates for rhabdomyosarcoma have included the RAS-signaling pathway, ALK, NTRK, FGFR, and MSI-High. In addition, fusion genes (e.g., *PAX3/7-FOXO1*), which play an important role in diagnostic and prognostic factors, are also being investigated as potential therapeutic targets as their underlying backgrounds are gradually becoming clear. This review summarizes the overall picture of the development of novel therapies for rhabdomyosarcoma and discusses the direction that should be taken in the future.

## Simple Summary

In the review, the epidemiology, prognostic factors and recent treatment strategies of rhabdomyosarcoma are summarized. Moreover, potential molecular targets under investigation and the challenges are also discussed.

## 1. Introduction

Rhabdomyosarcoma (RMS) is one of the histological subtypes of soft tissue sarcomas (STSs) and is morphologically classified as a small round-cell sarcoma. The annual incidence of STS is approximately 50 per 1,000,000 people, and RMS accounts for a few percent of all STSs; however, it accounts for a high proportion of the STSs occurring in children [1].

Based on the Surveillance, Epidemiology, and End Results (SEER) database in the U.S., pediatric and young adult STS patients (<20 years old) accounted for 5.6% of all STS patients in the U.S., and in that population, RMS amounted for the largest share (58.6% of STS patients aged <20 years had RMS); moreover, RMS was the only STS histological type for which the median patient age was under 20 years, at 15 years [2]. A study tracking the incidence rates of pediatric RMS over time from 1975 to 2005 in the SEER database reported that as the time progressed, the incidence of pediatric RMS cases, particularly those with a specific histological type (i.e., the alveolar type), appeared to increase [3]. In Europe, the incidence and age distributions of patients with RMS are similar to those of the U.S.; based on a Swiss registry’s data, RMS accounts for 3.4% of all STS cases [4]. In the European cancer database EUROCARE-6, the ages at RMS onset are concentrated almost entirely in pediatric and adolescent and young adult (AYA) patients, and RMS is more prevalent among younger individuals [5]. In Asia on the other hand, the incidence of RMS has been considered relatively low compared to Western countries [6]. For example, Japan’s recent registry data for patients enrolled in 2016–2019 revealed that RMS accounted for 2.7% (*n* = 639) of all 23,314 STS cases [7].

As a small round-cell sarcoma, RMS is sensitive to cytotoxic chemotherapy agents and radiotherapy, and multidisciplinary treatment strategies have been investigated since the 1970s; the proportion of RMS patients achieving cure has steadily increased year by year with treatment advances [8]. However, the prognosis of RMS patients who are not cured with the initial multidisciplinary therapy remains poor, and an effective salvage treatment strategy has not been established for recurrent cases.

Traditionally, treatment strategies for RMS have been investigated and selected based on a risk stratification that considers the histological subtype and clinical factors such as the patient’s age and the primary site. However, as knowledge regarding the pathology-based diagnosis and genetic abnormalities has increased, it has become recognized that RMS affects a more heterogeneous patient population than previously thought. It is imperative to investigate the genomic backgrounds of the refractory patient population and develop specific treatment strategies. Moreover, since RMS predominantly affects children and young adults, it is essential to enhance measures designed to prevent late complications and organ dysfunction even in cases in which a cure is achievable.

With this background, the established prognostic risk factors and the standard treatments for RMS are explained below first, and then challenges within each patient subgroup, emerging insights, and potential novel treatment strategies for the future are discussed.

## 2. The Traditional Risk Classification of RMS

This section presents the prognostic factors for RMS patients that have been identified in prospective clinical trials and/or large retrospective analyses. The current risk classification established by aggregating that information is then described.

### 2.1. Pathological Subtypes

As of the recent World Health Organization (WHO) classification’s 5th edition published in 2020, the RMS subtypes are the embryonal, alveolar, pleomorphic, and spindle cell/sclerosing types [1]. Each histological subtype has a characteristic peak onset age: the embryonal type is most common in children, the alveolar type is the most common in the AYA age group and older, and the pleomorphic type occurs most frequently in middle-aged and older adults. The sclerosing type can occur in both younger and older adults.

The pathology-based diagnosis of RMS based on morphological features is notoriously difficult, particularly for STSs including RMS, with a significant frequency of discordance even among specialists [9]. The use of artificial intelligence (AI) as an adjunct to diagnoses obtained via hematoxylin–eosin staining was recently proposed [10]. In addition, efforts to confirm diagnoses and identify subtypes by using objective markers such as immunostaining and fusion gene detection continue to advance. The *FOXO1* fusion gene in the alveolar subtype is particularly well known; since ~2000, it has been recognized that *PAX3-FOXO1* or *PAX7-FOXO1* fusion genes resulting from the fusion of *FOXO1* (formerly called *FKHR*) with either *PAX3* or *PAX7* are frequently observed in alveolar RMS, and their diagnostic value and potential as prognostic factors have been investigated [11]. FOXO1 is widely expressed and mediates signal-responsive transcriptional regulation in normal cells, but when fused with *PAX3* or *PAX7*, it becomes an oncogenic transcription factor with abnormally strong transcriptional activity in myogenic progenitor cells [12]. *PAX3-FOXO1* in particular had been considered a significant poor-prognosis factor [13,14], but with the accumulation of patient prognosis data, the distinction between *PAX3-FOXO1* and *PAX7-FOXO1* has recently become considered less critical [15]. In contrast, alveolar RMS that is fusion-gene-negative has been known to exhibit clinical sequences that are more similar to those of embryonal RMS than those of fusion-gene-positive alveolar RMS [16]. The classifications of embryonal RMS and alveolar RMS have thus been increasingly decided based on the presence or absence of fusion genes rather than morphological characteristics.

In spindle/sclerosing RMS, various rearrangements such as *VGLL2*, *CITED2*, *NCOA2*, *MEIS1*, *EWSR1*, and *TFCP2* can be observed; they might help the pathological diagnosis [1]. Recently, *MYOD1* mutations have been identified as an important prognostic factor in spindle/sclerosing RMS, which will be discussed in Section 4.1.

Pleomorphic RMS does not have the specific gene fusions or rearrangements necessary for diagnosis, and is considered as having gene profiles indistinguishable from undifferentiated pleomorphic sarcoma (UPS) [1].

### 2.2. Patient Ages

As noted above, RMS primarily affects children and young adults and occasionally adults. Comparisons of the prognosis of patients with RMS by their age at the onset of disease and using multiple registries’ data have consistently reported that adult-onset cases have a significantly worse prognosis compared to pediatric-onset cases [17,18]. However, RMS occurring in infants (<1 year old) or children ≥10 years old has been reported to have a relatively poor prognosis [19,20].

Differences in prognosis based on the patient’s age are influenced by factors such as variations in histological subtypes (embryonal RMS is more common in children, while other histological types are more frequent in adults) and tolerance to chemotherapy (relatively higher doses of anticancer drugs can be administered to children based on body weight). It is therefore difficult to pinpoint which factors directly correlate with prognosis. These age-related differences nevertheless remain important considerations when establishing overall prognosis and treatment strategies.

Spindle/sclerosing RMS can occur in both children and adults. While pediatric cases tend to have a favorable prognosis, adult-onset cases are associated with a poor prognosis.

Pleomorphic RMS exhibits a distinct clinical profile compared to other RMS subtypes: it affects adults aged ≥40 years—older than the AYA generation—with a higher age of onset and lower sensitivity to chemotherapy; consequently, it is often treated as a separate entity within RMS subtypes [21].

### 2.3. Primary Lesion

RMS shows a unique distribution of primary sites among the STSs, and it has been reported that the prognosis varies depending on the primary site. The head and neck region is one of the representative primary sites for RMS, and multiple registries’ data indicate that among primary head and neck sarcomas, RMS accounts for the highest proportion [22,23]. Primary head and neck RMS is generally considered to have a favorable prognosis, but parameningeal lesions tend to have a poor prognosis [24]. There is an ongoing debate regarding whether early administration of radiation therapy within an appropriate range can improve the prognosis of RMS [25,26,27]. There are also prognostic registry data for RMS located in other primary organs such as urological organs (kidney, bladder, prostate) and gynecological organs; of them, cases of genito-urinary non-bladder/prostate RMS were reported to have favorable prognoses [28,29]. Conversely, RMS originating in the extremities is associated with a poor prognosis. Recent results from Children’s Oncology Group (COG) clinical trials also indicate that the prognosis remains poor in these cases [30]. The biliary tract had been thought to be a favorable prognostic primary site, but the outcome of a COG clinical trial suggests that it may instead be associated with a poor prognosis [31].

It remains unclear whether pathological subtype or primary site has a greater impact on prognosis, and to what extent differences in pathological subtype affect prognosis even among patients with the same primary site. Further clarification is anticipated through future clinical trials and the accumulation of registry data.

### 2.4. Risk Stratification

**Figure 1:**
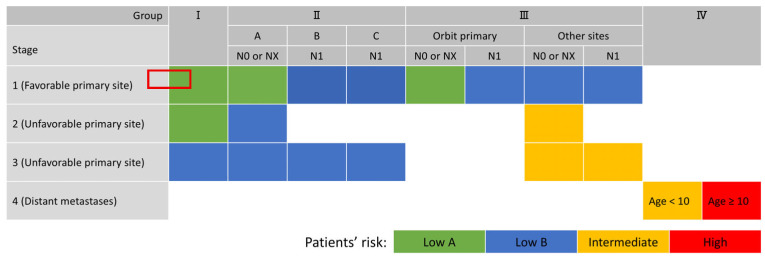
Risk stratification of embryonal RMS in IRS-V protocol.

Until recently, risk stratification for RMS patients had been performed by combining the established factors described above. The Intergroup Rhabdomyosarcoma Study (IRS), an international collaborative trial group, conducted a staging system and risk stratification for RMS (specifically for the embryonal type) based on the primary site, tumor size, presence/absence of lymph node metastasis, and presence/absence of distant metastasis. The results are summarized in Table 1 and Table 2 and depicted in Figure 1 [8]. Based on the grouping system, the estimated 5-year failure-free survival (FFS) rate was 90% for patients with low A, 87% with low B, and with intermediate 73%, respectively; in contrast, the 5-year FFS for the group III patients with stage 2 or 3 disease was 73%, the corresponding rate for the group III patients with stage 2 or 3 disease and T2 tumors who were <1 year or ≥10 years old was 56%, and that for group III patients with stage 2 or 3 disease and extremity primary tumors was 43% [32].

Improvements in the treatment of RMS have been pursued according to these risk categories. Note that for the alveolar type, cases without distant metastasis are uniformly classified as moderate risk, while cases with distant metastasis are classified as high risk because the risk stratification was established before the clinical significance of *FOXO1* fusion gene had been certified. The risk factor tables and figure have been in use, with minor adjustments made as clinical trial data have accumulated [33].

For patients with metastatic RMS, a prognostic prediction score based on patient age, primary tumor site, bone or bone marrow metastasis, and number of metastases, i.e., the Oberlin score, was proposed; the score stratification was derived from the patient backgrounds and clinical courses of 788 subjects across nine clinical trials conducted in Europe and the U.S. (Table 3). For each factor, one point is added if it corresponds to a poor prognosis, and a higher total score indicates a poorer prognosis. In the original patient group, the event-free survival (EFS) rate was 50% for patients without any of these four factors and was 42%, 18%, 12%, and 5% in patients with one, two, three, or four factors, respectively [34].

Subsequent prospective clinical trials and recent large-scale registry data have confirmed that the Oberlin score remains an effective prognostic scoring system for patients with metastatic RMS [35,36]. Regarding metastatic cases, the Oberlin score may be a more important prognostic factor than the presence/absence of *FOXO1* fusion gene in alveolar RMS [37].

For example, in the Frontline and Relapsed Rhabdomyosarcoma (FaR-RMS) clinical trial, currently underway by the European Paediatric Soft Tissue Sarcoma Study Group (EpSSG), a more detailed risk classification based on the IRS group classification is designed for evaluating patient prognosis and appropriate treatment interventions (Table 4) [38].

## 3. Current Risk-Based Treatment Strategies and Investigations

The RMS treatment strategies that have been established to date based on the risk assessment described above are discussed next. As stated in the Introduction, RMS generally demonstrates good sensitivity to chemotherapy and radiation therapy, and in principle, the standard treatment is multidisciplinary therapy combining multi-agent chemotherapy with local therapy (radiation therapy). However, unlike the other subtypes, the treatment of pleomorphic RMS that follows the strategy for non-small round-cell sarcoma is recommended.

**Figure 2:**
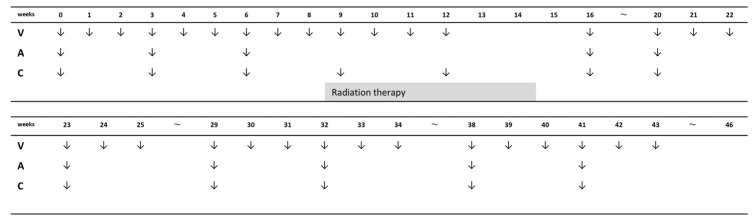
An example schedule of a vincristine, actinomycin-D, cyclophosphamide (VAC) regimen. A: actinomycin-D, C: cyclophosphamide, V: vincristine.

The standard chemotherapy regimen for RMS is the combination of vincristine (VCR), actinomycin-D (ACD), and cyclophosphamide (CPA), i.e., VAC therapy (Figure 2). This regimen is continued for nearly 12 months, interspersed with local treatment (radiation therapy) [39]. There are some protocols using ifosfamide instead of CPA as the alkylating agent [38].

Starting from this treatment schedule, adjustments such as adding or omitting drugs and modifying the treatment duration have been investigated based on the patient risk, which includes exploring adding new molecularly targeted drugs or modifying details of local therapies, particularly adjusting radiation therapy doses.

### 3.1. Low-Risk Patients

In low-risk patients, the conventional VAC protocol will achieve high cure rates; however, VAC includes high doses of CPA, which raises concerns about late toxicities such as infertility [40,41] and the risk of secondary malignancy [42]. Moreover, prolonged VAC treatment may impact a patient’s growth and educational achievement, thus affecting survivors’ quality of life [43]. Research is therefore underway to reduce the drug dose intensity (especially CPA, an alkylating agent) and shorten the treatment duration while maintaining the therapeutic efficacy [44,45]. Although the treatment efficacy and short-term prognosis are assured [46], concern that reducing the drug doses or treatment duration may increase the risk of recurrence has been described [47]. Consequently, the application of a reduced dose intensity requires the careful evaluation of both the patient population and the treatment content while confirming long-term prognosis outcomes. Adding irinotecan alongside a reduction in the CPA dose is also being considered as a means to increase the treatment intensity [48].

### 3.2. Intermediate-Risk Patients

For intermediate-risk patients, the addition of new drugs and/or treatments to a VAC protocol is being investigated as part of the effort to achieve higher cure rates. The RMS 2005 trial performed by the European Paediatric Soft Tissue Sarcoma Study Group (EpSSG) evaluated the efficacy of (i) adding doxorubicin (which has long been used as standard therapy for non-RMS STSs) to VAI therapy, and (ii) adding maintenance therapy with vinorelbine (VNL) and continuous low-dose CPA independently; the results demonstrated that adding doxorubicin did not provide an additional benefit, but adding VNL and CPA maintenance therapy improved the prognosis [49,50]. It is speculated that this improvement in prognosis may have resulted from controlling “tumor cells in a state close to dormancy” following standard treatment through the continuous administration of low-dose anticancer drugs.

### 3.3. High-Risk/Metastatic, Recurrent Cases

Since it remains challenging to achieve a cure with VAC therapy alone for metastatic or high-risk RMS cases, there are ongoing investigations of new multidisciplinary treatment strategies. Clinical trials incorporating a topoisomerase inhibitor such as topotecan or irinotecan into the initial therapy have demonstrated some efficacy, but sufficient effectiveness to replace VAC therapy has not been established [51,52]. The ARST0431 trial observed promising efficacy with a 3-year EFS rate at 38% and a 3-year overall survival (OS) rate at 56% by using highly intensive chemotherapy (ifosfamide/etoposide, vincristine/doxorubicin/cyclophosphamide, and irinotecan); however, efficacy data from randomized controlled trials are lacking, and due to significant adverse events, the clinical position of this intensive chemotherapy remains unestablished [53]. While the efficacy of high-dose chemotherapy and hematopoietic stem cell transplantation was once tested in clinical trials, meta-analyses have evaluated their effectiveness as limited, and clinical trials are no longer actively pursued [54]. Instead, the development of molecularly targeted therapies, as discussed in Section 4, has become the primary focus of treatment development for rhabdomyosarcoma.

No treatment with established efficacy is available as salvage therapy in cases of relapse after an initial treatment, and the prognosis of relapsed RMS patients remains dismal.

## 4. The Need for Novel Treatment Strategies and Drugs Based on the Current Risk-Based Treatment Strategies

### 4.1. Novel Risk Factors: MYOD1 Mutation

As our understanding of malignancies deepens, more detailed information has become available regarding RMS, including its traditional histological diagnosis and classification. A prime example is the MYOD1 mutation, identified in the 2010s as a new poor-prognosis factor for RMS.

The MYOD1 mutation was observed to be a poor-prognosis factor for embryonal RMS [55], and its presence has also been reported in spindle cell/sclerosing-type RMS [56,57]. Spindle/sclerosing RMS generally has a favorable prognosis in pediatric cases, but some cases with poor prognosis are known. Detecting MYOD1 mutations is highly effective for identifying these poor-prognosis cases [58]. Though the mechanism underlying the poor prognosis in MYOD1-mutated RMS remains unclear, immunohistochemical analysis of MYOD1-mutated RMS reveals strong upregulation of MYOD1 expression and reduced expression of MYF4 (a myogenin family transcription factor), potentially reflecting abnormalities in the skeletal muscle differentiation program. Furthermore, the p.L122R mutation, the most common in MYOD1-mutated cases, is known to reduce the normal target gene activation capacity of MYOD1, suggesting that normal differentiation induction is impaired [59]. Incorporating MYOD1 mutations into the RMS risk classification is also being considered, as is being done for the *FOXO1* fusion gene in alveolar RMS [60]. Research on MYOD1 mutations as a potential therapeutic target is also advancing [61]. In addition to MYOD1, new gene mutations and fusion genes may be incorporated into the criteria for determining RMS treatment options in the future; for example, the presence of new fusion genes such as *NCOA2-MEIS1*, *CAV1-MET*, *HMGA2-NEGR1*, and *RAB3IP-HMGA2* has also been reported in spindle/sclerosing RMS in recent years [62,63]. It is anticipated that the clinical significance as prognostic factors and potential as therapeutic targets of these newly discovered fusion genes will be further explored.

### 4.2. Molecular Testing/Comprehensive Genome Profiling (CGP)

The integration of data from cases enrolled in earlier clinical trials has also highlighted biases in patient backgrounds and risks across individual trials [64]. The importance of molecular testing and comprehensive genome profiling (CGP) is thus increasing in efforts to accurately perform patient background stratification and risk assessment. Although the potential for CGP to directly inform treatment selection and to improve patient prognoses remains under investigation, several research groups have suggested that molecular testing can contribute to risk assessments and prognosis improvement [65,66,67].

Establishing and maintaining a system for the comprehensive genomic analyses of RMS patients will be crucial to linking these findings to the development of the novel therapeutic targets presented next; CGP could enable the provision of molecularly targeted therapy to patients for whom it is expected to be effective, either as an addition to current standard treatment or as a new treatment for treatment-resistant cases.

Furthermore, in recent years, single-cell transcriptomic profiling has enabled more detailed evaluation and characterization of cellular states. Integrated analysis of single-cell RNA-seq data from pediatric RMS cells has revealed distinct properties between fusion-positive RMS and fusion-negative RMS [68]. Moving forward, intratumoral heterogeneity may be linked to tumor origin and treatment resistance, potentially providing clues for personalized therapeutic targets and strategies to overcome resistance.

### 4.3. Candidates for Novel Targeted Therapies for RMS

This section presents the status of genetic alternations that could become candidates for new therapeutic targets for RMS and the current development of treatments.

#### 4.3.1. The MAPK Pathway

The mitogen-activated protein kinase (MAPK) pathway is a cell proliferation pathway involving the direct phosphorylation cascade RAS/RAF-MEK-ERK, and it is known to harbor mutations in many solid tumors [69]. RAS mutations (including NRAS, KRAS, and HRAS) are also known to be present in approx. 20–30% of fusion-gene-negative cases of RMS (especially in embryonal RMS) [65,70,71].

As molecularly targeted therapies that have been designed to target the MAPK pathway, drugs that target downstream targets such as mTOR (mammalian target of rapamycin) and Akt are approved for breast cancer [72], renal cell carcinoma [73,74], and neuroendocrine tumors [75]. The development of the mTOR inhibitor temsirolimus, approved for renal cell carcinoma, has been investigated for the treatment of RMS. Based on the results of a Phase II study evaluating the use of temsirolimus in combination with cytotoxic chemotherapy for recurrent/metastatic cases of RMS [76], a Phase III trial (ARST1431) was conducted to assess the efficacy of adding temsirolimus to standard therapy for intermediate-risk RMS patients, but the results failed to show the superiority of adding temsirolimus [77]. However, these trials did not impose patient inclusion restrictions based on RAS mutations, and it is thus possible that these targeted therapies could be effective in specific patient populations. The efficacy of treating RMS by targeting MEK, which is further downstream of mTOR and the efficacy of combining MEK-targeting agents with other agents that inhibit upstream signaling pathways are currently being investigated, and promising data have been obtained in preclinical studies [78,79,80].

Although the development of molecularly targeted drugs that directly target RAS as a therapeutic target has long been challenging, several drugs have emerged in recent years that are entering the clinical setting. These drugs are already approved for KRAS G12C mutation-positive non-small-cell lung cancer (NSCLC) and colorectal cancer (CRC) [81,82,83,84], and their efficacy is being investigated for other cancer types [85]. Targeted RAS therapy is being evaluated across tumor types in a mutation-specific manner [86,87], and it is thus anticipated that reports of effective cases for RMS will emerge from ongoing clinical trials of RAS inhibitors.

#### 4.3.2. ALK

Anaplastic lymphoma kinase (ALK), which is also known as ALK tyrosine kinase receptor, has shown various alterations such as overexpression, mutation, and fusion protein formation; ALK contributes tumor progression, thereby functioning as a driver gene [88]. Approximately 5% of NSCLC cases are positive for ALK fusion genes, most commonly *EML4-ALK*. For ALK-positive NSCLC, many ALK-targeted small molecular targeted drugs have been investigated since the 2010s and have shown high response rates, resulting in the drugs’ approval [89]. The efficacy of ALK-targeted therapy has also been investigated for other malignant diseases that exhibit ALK alternations, focusing primarily on ALK fusion genes. Inflammatory myofibroblastic tumor (IMT) was observed to be particularly common among ALK-fusion-gene-positive patients with bone or soft tissue sarcomas, and clinical data demonstrating the efficacy of ALK-targeted therapy have been reported [90,91,92]. The approval of such therapies across different tumor types is anticipated [93].

ALK alterations are frequently observed in patients with RMS—particularly the alveolar subtype, which shows high positivity for ALK alterations on immunohistochemistry and high ALK gene copy number gain; high ALK expression has also been reported to correlate with poor prognosis. The PAX3/7-FOXO1 fusion protein observed in alveolar RMS is considered as a central driver of tumor initiation and progression, forming a transcriptional program that indirectly enhances the expression of several tumor-associated genes, including ALK [94]. However, a Phase II trial evaluating the efficacy of the ALK-targeted tyrosine kinase inhibitor crizotinib in RMS patients, including patients with alveolar RMS (EORTC90101; CREATE), did not demonstrate sufficient efficacy against RMS [95]. These negative results may have been obtained because ALK alterations in RMS do not act as tumor drivers like the fusion genes in other malignancies do, and/or because ALK inhibition alone is insufficient to halt tumor progression [96].

In recent years, ALK-fusion-gene-positive RMS cases such as those involving *ATIC-ALK* and *DCTN1-ALK* have been reported [97,98], and ALK-targeted therapy was effective in these cases. Other case reports described the observation of ALK activation in *FUS-TFCP2*-positive RMS, mainly in cutaneous primary-site cases [99,100], which suggests that ALK-targeted therapy may be effective for these patients. Although the ALK-targeted therapies approved to date for various cancers (such as NSCLC) are all small-molecule kinase oral agents, an ALK-targeted antibody–drug conjugate (ADC) is currently under development, and preclinical data suggest its promising efficacy for RMS [101]. It is anticipated that new ALK-targeted therapies that are effective against RMS will be identified and approved in the future.

#### 4.3.3. NTRK Fusion

The neurotrophic receptor tyrosine kinase (*NTRK)* gene encodes nerve growth factor receptors in healthy tissues and has three subtypes (*NTRK1*, *NTRK2*, and *NTRK3*). If combined with other genes, *NTRK* fusion genes activate, producing proteins that are involved in cell proliferation in a disorderly manner and resulting in tumor progression [102]. Although *NTRK*-fusion-gene-positive cancers are very rare, accounting for 1–2% of all solid tumors, it has been reported that tyrosine kinase inhibitors that have been approved as tumor-agnostic indications demonstrated extremely high efficacy against *NTRK*-fusion-gene-positive solid tumors [103,104]. Given this finding, the identification of appropriate patients is crucial. *NTRK*-fusion-positive cases are relatively more frequent in pediatric and AYA cancer patients than in older adults [105]. Soft tissue sarcomas in particular include histological types in which *NTRK*-fusion-positive genes are frequently detected, such as infantile fibrosarcoma and inflammatory myofibroblastic tumor (IMT) [106,107].

Although reports of *NTRK*-fusion-positive cases in RMS remain extremely limited, the case of an 11-year-old patient with *NTRK1*-fusion-gene-positive embryonal RMS was recently reported (a *MEF2D-NTRK1*-fusion-positive case) [108]. This patient did not receive treatment with an *NTRK* inhibitor, and the efficacy of *NTRK* inhibitors remains unclear. However, as noted above, *NTRK* inhibitors are tumor-agnostic agents, which indicates that promising targeted therapy options may be identified in future research. Major *NTRK* fusion genes are currently detectable by gene panel tests that are approved for routine clinical use (e.g., FoundationOne^®^ and GenMineTOP^®^), but some may not be detected depending on the partner gene. It is also important to update testing systems so that *NTRK* fusion gene patterns that are specifically associated with RMS can be detected by using test panels which are available for routine clinical practice, once the information about these patterns becomes clear.

#### 4.3.4. FGFR

Fibroblast growth factor receptor (FGFR) is a receptor tyrosine kinase that transmits signals into the cell upon binding with fibroblast growth factor (FGF), thereby regulating normal cellular functions. FGFR has four subtypes—FGFR1–4 [109]—and FGFR aberrant activations (mutation, overexpression, and gene fusion) are observed in various cancers. Targeted therapies are currently approved for FGFR2-fusion-gene-positive cholangiocarcinoma [110,111] and urothelial carcinoma with FGFR3 mutations [112]. Organ-transcending therapeutic agents are also being investigated [113,114].

FGFR4 alteration has been observed in approx. 10% of RMS cases; notably, the product of the *PAX3-FOXO1* fusion gene is known to induce FGFR4 expression, leading to high FGFR4 expression in alveolar RMS [115]. FGFR4 is thus considered a promising therapeutic target candidate for RMS. *FGFR1* fusion genes are also reported as potential drivers of RMS [116]. Preclinical studies have shown that certain FGFR inhibitors were effective against RMS cells harboring FGFR4 mutations, such as FGFR4 V550L [117,118]. FGFR4-mutant RMS cells have been reported to depend on heat shock protein 90 (HSP90), suggesting potential antitumor effects from HSP90 inhibitors. HSP90 inhibitors have been investigated primarily for prostate cancer [119], and the HSP90 inhibitor pimitespib recently demonstrated efficacy against gastrointestinal stromal tumor (GIST) and is approved in Japan [120]. Further data concerning the clinical efficacy of these inhibitors for the treatment of RMS is required. Chimeric antigen receptor T cell (CAR-T) therapy, a T-cell infusion treatment targeting FGFR, is also being investigated for RMS [121,122,123].

#### 4.3.5. MSI-High Status

Microsatellite instability (MSI)-High is an abnormality in which the number of repeats in short tandem repeats (microsatellites) on the genome changes due to dysfunction of the error-correction system during DNA mismatch repair (MMR). MSI-High is observed in Lynch syndrome (a hereditary cancer syndrome) and also sporadically observed in solid tumors at a low frequency. Patients with Lynch syndrome have mutations in MMR genes such as MLH1, MSH2, MSH6, and PMS2, which brings about the MSI-High status and increases the risk of multiple cancers; pleomorphic RMS has been reported as one histological type that can occur in sarcomas associated with Lynch syndrome [124]. As mentioned above, pleomorphic RMS has a recommended treatment regimen that is distinct from those used for other RMS subtypes. Its genomic landscape is also known to differ significantly from those of other RMS subtypes and is similar to those of UPS [125], with MSI-High cases reported primarily in patients with a Lynch syndrome background.

Immune checkpoint inhibitors have efficacy for MSI-High tumors and have been approved for the treatment of solid tumors as tumor-agnostic agents [126]. Several case reports describe treatment with immune checkpoint inhibitors for MSI-High pleomorphic RMS, in patients with Lynch syndrome or in sporadic cases [127,128,129]. Going forward, evaluating the prevalence of MSI-High cases among patients with pleomorphic RMS may provide an opportunity to explore the necessity of MSI-High screening and the efficacy of immune checkpoint inhibitors. It is also hoped that new immunotherapies other than immune checkpoint inhibitors will be developed in the future.

#### 4.3.6. CDK4

Cyclin-dependent kinase 4 (CDK4) is a cyclin-dependent kinase essential for the transition from the G1 to the S phase of the cell cycle, and in rhabdomyosarcoma, particularly *PAX3-FOXO1*/*PAX7-FOXO1*-positive cases, gene amplification in the 12q13-q14 region is frequently observed. There is a preclinical study that shows that CDK4 is overexpressed due to 12q13-q14 amplification in fusion-gene-positive RMS and that CDK4 knockdown suppresses tumor cell proliferation and transformation potential [130]. Additionally, cyclin-dependent kinase CDKN2A inhibits CDK4/6 activity and encodes p16INK4A and p14ARF. It is reported that loss of p16INK4A in RMS cells with CDKN2A deletion/mutation enhances CDK4/6 activity, leading to tumor proliferation in preclinical studies [131].

CDK4 inhibitors are currently approved for breast cancer [132], and as for STSs, there are clinical trials for liposarcoma [133]. Reports suggest that CDK4/6 inhibitors may demonstrate tumor-suppressive effects in vivo against fusion-positive RMS, raising expectations for their potential as future therapeutic candidates [130].

#### 4.3.7. Epigenetic Therapeutic Targets

Epigenetic variations that regulate gene expression are known to be involved in the onset and progression of cancers, including RMS, and may serve as therapeutic targets.

Enhancer of zeste homolog (EZH2) is gaining attention as a candidate epigenetic therapeutic target for RMS. EZH2 is a catalytic subunit of the PRC2 complex, responsible for adding H3K27me3, thereby suppressing gene expression and preventing differentiation in undifferentiated myoblasts. In RMS, EZH2 is overexpressed, leading to suppressed muscle differentiation and the proliferation and progression of undifferentiated tumors [134]. Preclinical studies have reported that inhibiting EZH2 suppresses the proliferation of RMS tumor cells [135], raising expectations that the efficacy of EZH2-targeted therapy will be evaluated in clinical settings in the future.

MicroRNAs are also candidates for cancer drivers at the microscopic level. In RMS, miRNAs essential for normal muscle differentiation are known to show abnormal expression, suggesting their involvement in tumorigenesis and differentiation inhibition [136]. Among these, the miR-29 family has been shown to suppress proliferation and invasion in RMS cells, promote apoptosis, and reduce tumorigenicity; this indicates that miR-29 family members may act not merely as biomarkers, but as drivers in RMS that function as regulatory factors controlling fundamental properties of tumor cells [137].

## 5. Conclusions

Multidisciplinary treatment based on the results of past clinical trials is currently applied for RMS, but the prognosis for metastatic cases and treatment-resistant cases remains poor, necessitating the development of new therapeutic strategies. Furthermore, since RMS occurs frequently in pediatric and young adult patients, awareness and social support regarding late toxicity during long-term follow-up after treatment and survivorship are also important [138]. Given that RMS is a rare disease with a limited number of patients, it is essential to advance personalized medicine in order to accurately assess the prognosis for each individual case and provide appropriate treatment.

## Figures and Tables

**Table 1:** The Intergroup Rhabdomyosarcoma Study Group (IRSG) surgical and pathologic grouping system for RMS.

Group	Definition
I	Localized tumor, completely removed with pathologically clear margin and no regional lymph node involvement
II	Localized tumor, grossly removed with (a) microscopically involved margins, (b) involved, grossly resected regional lymph nodes, or (c) both
III	Localized tumor, with gross residual disease after grossly incomplete removal, or biopsy only
IV	Distant metastases present at diagnosis

**Table 2:** The IRSG staging system for RMS.

Stage	Sites of Primary Tumor	Tumor Size, cm	Regional Lymph Nodes	Distant Metastases
1	Orbit, non-parameningeal head/neck; genito-urinary non-bladder/prostate; biliary tract	Any size	N0, N1	M0
2	All other sites	≤5	N0	M0
3	All other sites	≤5	N1	M0
		≥5	N0 or N1	
4	Any site	Any size	N0 or N1	M1

**Table 3:** Oberlin score: risk stratification for metastatic RMS.

Parameter	Unfavorable Factor
Patient age	≤1 or ≥10
Primary tumor site	Limb and other-than-favorable sites ^1^
Bone or bone marrow involvement	Present
No. of metastatic sites	≥3

^1^ Favorable sites include: orbit, non-PM, PM, bladder/prostate, paratesticular/vagina.

**Table 4:** The risk stratification set in the FaR-RMS clinical trial.

Risk Group	Subgroup	Fusion Status	IRS Group	Site	Nodal Status	Size or Age
Low risk	A	Negative	I	Any	N0	Both favorable
Standard risk	B	Negative	I	Any	N0	One or both favorable
	C	Negative	II, III	Favorable	N0	Any
High risk	D	Negative	II, III	Unfavorable	N0	Any
	E	Negative	II, III	Any	N1	Any
	F	Positive	I, II, III	Any	N0	Any
Very high risk	G	Positive	II, III	Any	N1	Any
	H	Any	IV	Any	Any	Any

Fusion status: Where fusion gene status is unavailable, histopathology will be used. Non-alveolar disease should be defined as fusion-gene-negative, and alveolar disease should be defined as fusion-gene-positive. Site: Favorable sites are GU, including bladder–prostate, head and neck non-parameningeal, orbit and biliary primaries. Unfavorable sites are all other sites. Node stage: N0 = 0 positive lymph nodes and N1 = ≥ positive lymph node. Age: Favorable is defined as age over 1 and under 10 years of age at diagnosis. Size: Favorable primary tumor is ≤5 cm as the longest diameter, and patients that are assessed as not evaluable will be included in >5 cm group.

## Data Availability

No new data were created or analyzed in this study. Data sharing is not applicable to this article.

## Abbreviations

ACD	actinomycin-D
ADC	antibody–drug conjugate
ALK	anaplastic lymphoma kinase
AYA	adolescent and young adult
CAR-T	chimeric antigen receptor T cell
CGP	comprehensive genome profiling
COG	Children’s Oncology Group
CPA	cyclophosphamide
CRC	colorectal cancer
EFS	event-free survival
EpSSG	European Paediatric Soft Tissue Sarcoma Study Group
FFS	failure-free survival
EZH2	enhancer of zeste homolog 2
FGF	fibroblast growth factor
FGFR	fibroblast growth factor receptor
GIST	gastrointestinal stromal tumor
HSP90	heat shock protein 90
IMT	inflammatory myofibroblastic tumor
IRS	Intergroup Rhabdomyosarcoma Study
MAPK	mitogen-activated protein kinase
MSI	microsatellite instability
mTOR	mammalian target of rapamycin
NSCLC	non-small-cell lung cancer
NTRK	neurotrophic receptor tyrosine kinase
OS	overall survival
RAS	renin–angiotensin system
RMS	rhabdomyosarcoma
SEER	Surveillance, Epidemiology, and End Results
STS	soft tissue sarcoma
UPS	undifferentiated pleomorphic sarcoma
VCR	vincristine
VNL	vinorelbine
